# Steps toward a digital twin for functional food production with increased health benefits

**DOI:** 10.1016/j.crfs.2023.100593

**Published:** 2023-09-26

**Authors:** Helena Mylise Sørensen, David Cunningham, Rengesh Balakrishnan, Susan Maye, George MacLeod, Dermot Brabazon, Christine Loscher, Brian Freeland

**Affiliations:** a School of Biotechnology, Dublin City University, D9 Dublin, Ireland; b I-Form, Advanced Manufacturing Research Centre, Dublin City University, D9 Dublin, Ireland; c Dairygold Co-Operative Society Limited, Clonmel Road, Co. Cork, P67 DD36, Mitchelstown, Ireland

**Keywords:** Digital twin, Bioprocessing, Process control, Process modelling, Artificial neural network

## Abstract

*Lactobacillus rhamnosus* (*L. rhamnosus*) is a commensal bacterium with health-promoting properties and with a wide range of applications within the food industry. To improve and optimize the control of *L. rhamnosus* biomass production in batch and fed-batch bioprocesses, this study proposes the application of artificial neural network (ANN) modelling to improve process control and monitoring, with potential future implementation as a basis for a digital twin.

Three ANNs were developed using historical data from ten bioprocesses. These ANNs were designed to predict the biomass in batch bioprocesses with different media compositions, predict biomass in fed-batch bioprocesses, and predict the growth rate in fed-batch bioprocesses.

The immunomodulatory effect of the *L. rhamnosus* samples was examined and found to elicit an anti-inflammatory response as evidenced by the inhibition of IL-6 and TNF-α secretion.

Overall, the findings of this study reinforce the potential of ANN modelling for bioprocess optimization aimed at improved control for maximising the volumetric productivity of *L. rhamnosus* as an immunomodulatory agent with applications in the functional food industry.

## Highlights

• Development of building blocks to operationalize a digital twin for fed-batch bioprocesses using *Lactobacillus rhamnosus.*
• Utilization of artificial neural network (ANN) modelling to optimize process control and monitoring.
• Prediction of biomass and growth rate in batch and fed-batch bioprocesses using ANNs.
• Examination of the immunomodulatory effects of *Lactobacillus rhamnosus* samples.

## Graphical abstract

**Figure:**
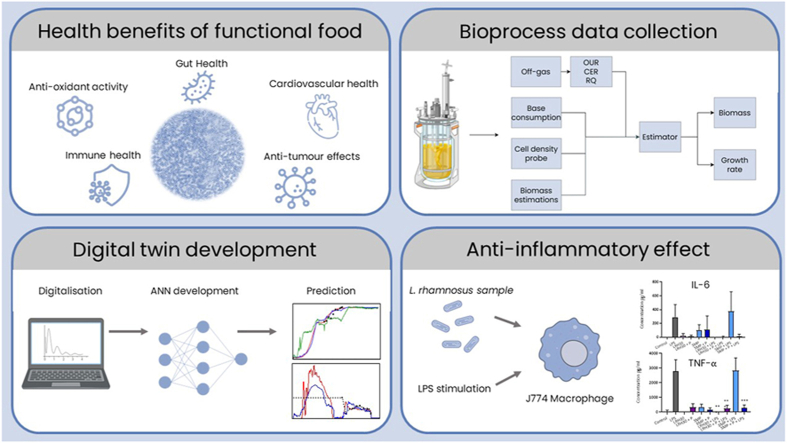

## Nomenclature

F_0_: Initial feed rate (ml/h)
λ_x_: Delayed time variable of Lactobacillus (h)
μ: Growth rate (h^−1^)
S_i_: Substrate concentration in inlet medium (limiting substrate) (kg/m^−3^)
V_x_: Volumetric cell growth rate (g/L/h)
X_m_: Maximum cell concentration (g/L)
Y_x/s_: Yield coefficient of biomass per substrate, incl. Maintenance (kg/kg^−1^)
ANOVA: Analysis of variance
ANN: Artificial Neural Network
AU: Absorbance units
CER: Carbon evolution rate
CDW: Cell dry weight
ELISA: Enzyme linked immunosorbent assay
FBS: Fetal bovine serum
HRP: Horseradish peroxidase
J774: J744A.1 murine macrophage
IL: Interleukin
LAB: Lactic Acid bacteria
LPS: Lipopolysaccharide
LRH30: *Lactobacillus rhamnosus LRH30*
MLR: Multiple linear regression
MRS: Man, Rogosa and Sharp
MTS: 3-(4,5-dimethylthiazol-2-yl)-5-(3-carboxymethoxyphenyl)-2-(4-sulfophenyl)-2H-tetrazolium
MSE: Mean squared error
NIR: Near infrared
OUR: Oxygen uptake rate
RD: Reagent dilutent
RMSE: Root mean squared error
RPM: Revolutions per minute
RQ: Respiratory quotient
SE: Standard error
SMP: Skim milk powder
TCD: Total cell density
TMB: 3,3′,5,5′-tetramethylbenzidine substrate
TNF: Tumour necrosis factor

## 1 Introduction

The ultimate goal of bioprocess development is achieving maximum productivity while meeting quality demands as well as ensuring process sustainability. Traditionally these requirements have been achieved by following a standard approach; through microbial strain engineering, media alteration and optimizing the extrinsic fermentation parameters (Makino et al., 2011; Wang et al., 2020; Haj-Mustafa et al., 2015). While these methodologies are indeed still relevant, the movement towards better automation in biomanufacturing underlines the need for continuous development of process engineering tools, specifically within process modelling, estimation, and control (Luo et al., 2021). As the markets of functional food ingredients continue to grow and evolve, the industry must keep investing and implementing these tools, to remain efficient and capable of meeting consumer demands (Rathore et al., 2021; Narayanan et al., 2020).

An important group of industrially relevant microorganisms are the lactic acid bacteria (LAB) which are widely applied within the food industry, particularly with dairy products. They contribute with not only flavour, texture and shelf-prolonging properties, but also impart health benefits to functional and fermented foods. This is accomplished both by the whole cells and the metabolites or postbiotics they excrete in their surroundings (Sørensen et al., 2022). The consumption of functional foods containing LAB or their postbiotics has shown to be beneficial to human health by having immunostimulatory effects, improving cardiovascular health, stimulating gut health and having antioxidant and antitumor effects (Liu et al., 2011; London et al., 2014; Vinderola et al., 2006; Namdari and Nejati, 2016; Zhang et al., 2013). These benefits are attractive to consumers, and the functional food market is estimated to experience an annual growth rate of 4.5%, reaching a market size of 219.5 billion US dollars by 2026 (Sørensen et al., 2022). With these production demands, it is essential for the industry to increase productivity by implementing process optimization tools along with the Manufacturing 4.0 frameworks to meet the increasing demands and complexity of this growing market.

Modelling is an essential tool in process optimization, quality-by-design and Manufacturing 4.0 approaches for bioprocess development. In upstream bioprocessing, the application of mathematical models to biological systems is used to simulate and optimize the growth of microorganisms (Tsopanoglou et al., 2021). The traditional modelling approach is through the use of mechanistic models using first principle models such as mass balances or empirical models such as the Monod equation (Mears et al., 2017a). Mechanistic models are widely applied due to their high level of accuracy when making predictions, but they require extensive experimental knowledge, are time-consuming to develop and are more difficult to apply to complex bioprocesses with several variables and interactions (Tsopanoglou et al., 2021; Del Rio-Chanona et al., 2019). Another type of modelling frequently applied is data-driven models and machine learning techniques (Del Rio-Chanona et al., 2019). As opposed to the mechanistic type models, this approach does not require in-depth knowledge of the physical process and is better able to handle large and complex datasets (Fung Shek et al., 2021; Zhang et al., 2019). Data-driven models do not however give insight into causal effects between variables, as they focus on the relationship between input and output variables. Hybrid models combining strengths from mechanistic models and data-driven models can therefore be more suitable in bioprocess engineering (Tsopanoglou et al., 2021).

Accurate estimations can be performed on certain variables of the bioprocess using sensors monitoring measurable variables such as temperature, dissolved oxygen, pH, and turbidity. More complex process variables such as biomass, metabolites and by-products are more challenging to directly estimate online. These will therefore often require the application of a soft sensor that uses mathematical models to confer the unmeasurable variable based on known process variables (Kornecki and Strube, 2019; Spann et al., 2018).

With both accurate bioprocess modelling tools, it is possible to obtain effective control of the process, ensuring robust manufacturing processes capable of meeting the quality needs with high levels of repeatability. Effective process control is also important for enhancing cell productivity by supporting cell proliferation and also maintaining and manipulating cell behaviour (Luo et al., 2021).

Artificial neural networks (ANN) are a type of data-driven model and machine learning algorithm that is inspired by the principles behind a biological nervous system and are comprised of layers of interconnected neurons that process information (Thibault et al., 1990; Sewsynker-Sukai et al., 2017). The basic topology of an ANN consists of an input layer, one or more hidden layers and an output layer, with each node connected by an associated weight. ANNs are a powerful tool for solving complex challenges. They offer several advantages in bioprocess engineering as an ANN can learn from historical data as well as interpret complex and non-linear relationships between inputs and outputs without prior knowledge of the structural relationship between these variables (Sewsynker-Sukai et al., 2017; Desai et al., 2005). These advantages have led to the successful application of neural networks in bioprocess engineering including in process optimization (Tholudur and Ramirez, 1996; Vlassides et al., 2001), for the prediction of fermentation variables (Thibault et al., 1990; Valdez-Castro et al., 2003) and for use in model predictive control (Nagy, 2007; Kiran and Jana, 2009).

The development of an ANN creates a prediction model of the bioprocess that gathers the information from the physical process that can then make predictions on the desired outcome. This can be applied as a component in the implementation of a digital twin. Here, the physical process communicates with a digital model of the process that simulates and optimizes the growth conditions which it then feeds back into the physical process. This allows for closed-loop control and monitoring of the process. This has been investigated in other studies and has seen implementations in feed rate control in fed-batch bioprocesses with real-time measurements of OUR, CER and base addition (Mears et al., 2017b), or real-time measurements of volatile off-gas analysis (Wegerhoff and Engell, 2016).

In this work, three ANNs were developed based on bioprocess data from the cultivations of *L. rhamnosus* in dairy-based media: one for the prediction of biomass in batch bioprocess with different feedstocks, one for biomass prediction in fed-batch bioprocess and finally an ANN for growth rate prediction during the fed-batch bioprocess. The development of data-driven predictive models is of significant importance for their integration as optimization and control tools in bioprocesses, with the potential to enhance efficiency and sustainability in production.

## 2 Materials and methods

### 2.1 Bacterial strain and growth conditions

*Lentilactobacillus rhamnosus LRH30* was provided by Sacco System (Cadorago, Italy). Stock culture of *L. rhamnosus* in a volume of 2 μL was precultured in 200 ml sterile (121 °C for 20 min) de Man, Rogosa and Sharp (MRS) broth (Oxoid, United Kingdom) in a conical flask for 24 h at 37 °C and 100 RPM. The inoculum for bioreactor fermentations was prepared by collecting the cell pellet from the preculture by centrifugation for 5 min at 5000 g and then resuspended to a total volume of 10 ml.

### 2.2 Media and feed

For the batch cultivations of *L. rhamnosus* the media consisted of a selection of different dairy-based media types in concentrations varying between 15 and 20 g/L: alpha-D-lactose (Acros Organics, Belgium), whey permeate (Volac International ltd, United Kingdom), demineralised whey (Dairygold, Ireland) or skim milk powder (Dairygold, Ireland). In addition, 4–5 g/L of yeast extract and 2–10 g/L of peptone (Thermo Fischer Scientific, Massachusetts, USA) and a salt solution consisting of 5 g/L sodium acetate anhydrous (Thermo Fischer Scientific, Massachusetts, USA), 2 g/L ammonium citrate (Alfa Aesar, Massachusetts, USA), 2 g/L KH_2_PO_4_ (Thermo Fischer Scientific, Massachusetts, USA), 0.2 g/L MgSO_4_ (Honeywell, Indiana, USA) and 0.04 g/L MnSO_4_ (Thermo Fischer Scientific, Massachusetts, USA), 1 g/L tween 80 (Thermo Fischer Scientific, Massachusetts, USA) and 2 ml of antifoam (Murphy & Son, Ireland) (Table 1). For fed-batch cultivations, all media consisted of 37 g/L skim milk powder, 14 g/L yeast extract 1 g/L tween 80, 2 ml of antifoam and the same salt solution used in the batch media (Table 1). For all media, carbon sources were autoclaved separately from the rest of the media, and both were autoclaved at 110 °C for 10 min to avoid the formation of milk gels. The feed was prepared from skim milk powder to get a total concentration of lactose of 100 g/L (Dairygold, Ireland).

**Table 1:** Media composition of bioprocesses and inoculum sizes. Batch bioprocess runs 1–5 were used as training datasets while run 6 was used as a testing dataset. Fed-batch bioprocess runs 7–9 were used as training datasets while run 10 was used as a testing dataset.

Media component (g/L)	Batch bioprocess run number						Fed-batch bioprocess run number			
	1	2	3	4	5	6	7	8	9	10
Lactose	20	–	–	15.23	–	–	–	–	–	–
Whey permeate	–	20.60	–	–	–	–	–	–	–	–
Demineralised whey	–		–	–	20.79	–	–	–	–	–
Skim milk powder	–	–	28.67	–	–	37	37	37	37	37
Yeast extract	4	4.15	4.15	4.15	4.15	14	14	14	14	14
Peptone	10	2.44	4.15	4.15	3.34	–	–	–	–	–
Tween 80	1	1	1	1	1	1	1	1	1	1
Sodium acetate	5	5	5	5	5	5	5	5	5	5
Ammonium citrate	2	2	2	2	2	2	2	2	2	2
KH_2_PO_4_	2	2	2	2	2	2	2	2	2	2
MgSO_4_	0.2	0.2	0.2	0.2	0.2	0.2	0.2	0.2	0.2	0.2
MnSO_4_	0.04	0.04	0.04	0.04	0.04	0.04	0.04	0.04	0.04	0.04
Inoculum size	0.2	0.08	0.04	0.06	0.11	0.4	0.39	0.45	0.48	0.4

### 2.3 Bioreactor setup

Stock cultures of *L. rhamnosus* were precultured in a volume of 2 μL in 200 ml sterile (121 °C for 20 min) MRS broth in screw-capped conical flasks for 24 h at 37 °C at 100 RPM. The cells were collected by centrifugation for 5 min at 840×*g* and resuspended to a total volume of 10 ml.

*L. rhamnosus* was precultured at 37 °C in a 3.6 L bioreactor with a working volume of 2.5 L (KLF, BioEngineering AG, Wald, Switzerland). All cultivations were maintained at pH 6 (±0.2) by the addition of either 3 M NaOH or 0.5 M H_2_SO_4_ utilizing a peristaltic pump connected to the computer. Agitation was set at 200 rpm to ensure complete mixing of the broth and an airflow rate was kept at 12 L/h and supplied through a sintered sparger (Wang et al., 2020). Temperature, pH and dissolved oxygen were monitored through probes (Mettler-Toledo, Columbus, USA) and controlled by software supplied by Bioengineering. Total cell density (TCD) was monitored through a near-infrared NIR probe (Exner, United Kingdom). Samples were withdrawn at intervals and kept at 5 °C for offline analysis.

The addition of feed was controlled by a Peripex W2 pump (Bioengineering AG, Wald, Switzerland) with the pump speed controlled by a LabVIEW-based controller (National Instruments, Texas, USA). The concentration of CO_2_ and O_2_ in the off-gas was monitored by a Tandem Pro gas analyser (Magellan Biotech, Borehamwood, United Kingdom).

### 2.4 Biomass determinations

The biomass of offline samples was determined by cell dry weight (CDW). Aliquots of bacterial cell suspensions were taken at the end of fermentation and were centrifuged at 3360×*g* for 10 min. Cell pellets were washed twice in distilled water and dried overnight at 80 °C. The dried cell pellet was then weighed to determine the bacterial CDW.

The biomass was monitored online by a TCD probe measuring the optical density of the fermentation broth at 880 nm. This signal was related to CDW offline by a calibration curve (Fig. S1).

### 2.5 Feed rate determination

An exponential feed strategy was applied for the fed-batch cultivations. Pre-determined growth rate values of 0.14, 0.19, 0.24 and 0.34 were utilized to control the feed.

The feed rate is time-dependent and can be described by Equation (1) where *t* denotes the time (h) and *F*_*0*_ is the initial feed rate (ml/h).

Equation 1 $$F\left(t\right)=F_{0}∙e^{\mu t}$$

This can be rewritten as:

Equation 2 $$F_{0}=\frac{\mu }{S_{i}∙Y_{x/s}}∙\left({X_{0}V}_{0}\right)∙e^{\mu t}$$

Where S_i_ is the substrate concentration in inlet medium (limiting substrate) (kg/m^−3^), Y_x/s_ is the yield coefficient of biomass per substrate, incl. Maintenance (kg/kg^−1^), X_0_ is the initial biomass concentration (kg/m^−3^) and V_0_ is the bioreactor volume at feed start (L) (Ding and Tan, 2006).

### 2.6 Logistic modelling of biomass

The logistic equation represents classic kinetic model techniques during batch cultivations that describe biomass development. A logistic model considering only cell growth variables and therefore independent of the substrate was applied to predict the biomass and is given in Equation (3) (Balakrishnan et al., 2020).

Equation 3 $$x=\frac{x_{m}}{1+\exp\left(2+\frac{4v_{x}}{x_{m}}∙\left(\lambda_{x}-t\right)\right)}$$

Where X_m_ describes the maximum cell concentration (g/L), λ_x_ is the delayed time variable of *Lactobacillus* (h) and V_x_ is the volumetric cell growth rate (g/L/h). This was fitted to the offline values of CDW by reducing the squared error (SE) between offline and predicted values in Equation (4).

Equation 4 $$SE={\left(y_{i}-\hat{y_{i}}\right)}^{2}$$

### 2.7 Specific growth rate estimation

The specific growth rate was calculated for the fed-batch cultivations by using the reconciled and filtered data and is given in Equation (5), where μ is the specific growth rate, X is the concentration of cells and V is the volume in the bioreactor, taking the increase of volume by feed and base addition during cultivation into consideration.

Equation 5 $$\mu \left(t\right)=\frac{dln\left(X∙V\right)}{dt}$$

To reduce the noise in the signal, a window of 20 min was selected, and an estimation of μ could be calculated using Equation (6):

Equation 6 $$\mu_{est}=\frac{\ln\left(\frac{x_{t}\cdot v_{t}}{x_{t-20}\cdot v_{t-20}}\right)}{\Delta t}$$

### 2.8 Off-gas analysis

The oxygen uptake rate (OUR), the carbon dioxide evolution rate (CER) and the respiratory quotient (RQ) were calculated post-fermentation by Equation (7), Equation (8) and.

Equation (9) (Hoppe et al., 2011).

Equation 7 $$OUR=\frac{\rho ∙M_{O2}}{R∙T}\left(F_{Air,in}∙\frac{C_{O,cal}}{100}-F_{Air,out}∙\frac{C_{O2,out}}{100}\right)$$

Equation 8 $$CER=\frac{\rho ∙M_{CO2}}{R∙T}\left(F_{Air,out}∙\frac{C_{CO2,out}}{100}-F_{Air,in}∙\frac{C_{CO,cal}}{100}\right)$$

Equation 9 $$RQ=\frac{CER}{OUR}$$

### 2.9 Data reconciliation

Data recorded at 5-min intervals constitute the final data sets. The data describing OUR, CER, RQ, absorbance units (AU) and growth rate all underwent data reconciliation by moving point average and median filtering with a selected time window of 20 min to minimize the effect of delay on the signals whilst ensuring a smoother dataset.

### 2.10 Artificial neural network development

An artificial neural network (ANN) is a computational modelling tool based on the concept of neural communication within the brain (Tarca et al., 2007). It utilizes historical data to understand the relationship between the input variables to perform a nonlinear prediction of the output variable. The development of an optimal neural network architecture is highly determined by the selection of the following hyperparameters: input layer, hidden layer and output layer. In addition, the architecture of an ANN is also dependent on activation functions and a number of hidden nodes in the hidden layer. For this study, the ANS Machine Learning Studio ANNHUB software was used for ANN development (ANS Center, Australia).

All conditions of the cultivations with relevance to the output variable were included as numerical input variables. Three models were developed namely.

1. ANN for biomass prediction in batch mode.
2. ANN for biomass prediction in fed-batch mode.
3. ANN for growth rate prediction in batch & fed-batch mode.

Input parameters for the prediction models were carefully selected, encompassing commonly used bioprocess parameters.

For the prediction of cell biomass in batch cultivations, the following input parameters were selected: Lactose concentration (g/L), total carbon solids (g/L), total solids (g/L), inoculum size (g/L), dissolved oxygen (%), accumulative base addition (ml), TCD (AU), OUR, CER and the respiratory quotient (RQ), with the output variable assigned as biomass (g/L). As the media was a constant variable for the fed-batch cultivations, the input variables describing media composition were removed.

For biomass prediction in fed-batch mode, the following input variables were selected: inoculum size (g/L), dissolved oxygen (%), accumulative base addition (ml), TCD (AU), OUR, CER, RQ and bioreactor volume (L) account for the addition of feed.

**Fig. 1:**
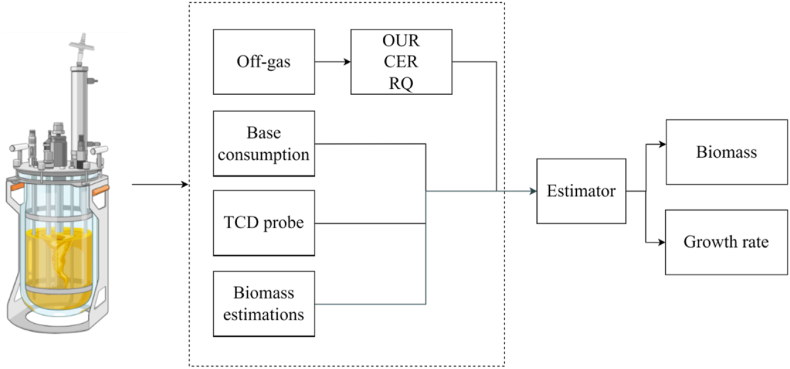
Overview of process from fermentation, data collection and development of estimator for biomass and growth rate.

After the successful development of an ANN prediction of biomass for fed-batch, the predicted value of biomass was used as an input parameter for the development of an ANN prediction of cell growth rate (μ), in addition to previously used input variables. A simple overview of the biomass and growth rate estimator can be seen in Fig. 1.

After the selection of input and output parameters, the data was normalized between 0 and 1 and divided into 3 sub-sets; 75% as the training data set, 12.5% as the validation data set and 12.5% as the testing data set. The performance of three training engines (Scaled Conjugate Gradient, Levenberg-Marquardt and Bayesian Regulation) were evaluated (Mia and Dhar, 2016). Hidden nodes were tested in a range between 1 and 15 to find the optimal size for the hidden layer.

In this study the *tansig* function was applied to process the hidden layer as described in Equation (10):

Equation 10 $$f\left(x\right)=\frac{2}{1+e^{-2x}}-1$$

For the processing of the output layer, the *logsig* function was applied Equation (11):

Equation 11 $$f\left(x\right)=\frac{1}{1+e^{-x}}$$

The cost function used in this study was a mean squared error (MSE) Equation (12):

Equation 12 $$MSE=\frac{1}{n}\sum_{i=1}^{n}{\left(y_{i}-\hat{y_{i}}\right)}^{2}$$

As evaluation metrics for the model developments root mean squared error (RMSE) (Equation (13)) and the coefficient of determination R^2^ were applied (Equation (14)):

Equation 13 $$RMSE=\sqrt{\frac{1}{n}\sum_{i=1}^{n}{\left(y_{i}-\hat{y_{i}}\right)}^{2}}$$

Equation 14 $$R^{2}=\frac{\sum {\left(y_{i}-\hat{y_{i}}\right)}^{2}}{\sum {\left(y_{i}-\bar{y_{i}}\right)}^{2}}$$

### 2.11 Sensitivity analysis of input variables

The relative effect of each of the input variables on the output variable in the neural networks was assessed through Equation (15):

Equation 15 $$v=\frac{\sum_{j=1}^{n_{H}}\left[\left(i_{vj}/\sum_{k=1}^{n_{v}}i_{kj}\right)O_{j}\right]}{\sum_{i=1}^{n_{v}}\left[\sum_{j=1}^{n_{h}}\left[\left(i_{vj}/\sum_{k=1}^{n_{v}}i_{kj}\right)O_{j}\right]\right]}$$

Where *v* is the relative effect of input variables, *n*_*H*_ is the number of neurons in the hidden layer, *i*_*vj*_ is the connection weight between the input neurons *i* and the hidden neurons *j*, *n*_*v*_ is the number of input variables, $\sum \frac{n_{v}}{k=1}$ *i*_*kj*_ is the sum of connection weights between input neurons *n* and hidden nodes *j*. *O*_*j*_ represents the absolute value of the connection weights between the hidden and output layer (Chellam, 2005).

### 2.12 Growth of J774A.1 murine macrophage cell stocks

Stocks of J744A.1 murine macrophage (J774) (European Collection of Authenticated Cell Cultures, Salisbury, United Kingdom) was maintained in Dulbecco's modified Eagle's medium (DMEM) (Fisher Scientific, New Hampshire, USA) with 10% heat-inactivated fetal bovine serum (FBS) (Biosciences, Dublin, Ireland) and 1% Pen-strep antibiotic (Biosciences, Dublin, Ireland). J774s were cultured in T75 flasks (Fisher Scientific, New Hampshire, USA) at 37 °C. Cells were passaged every three to four days by scraping J774s into existing media, then collecting cell suspension by centrifugation at 48×*g* for 5 min and resuspending in 10 ml of DMEM before finally adding 1 ml of resuspended cells to T75 flasks containing 30 ml DMEM media.

A 96-well microtiter plate (Thermo Fisher Scientific, Massachusetts, USA) containing 250 μL of J774 cells in a concentration of 10^6^ cells/ml were incubated at 37 °C in a humidified, 5% CO2 atmosphere for 24 h. Samples harvested from a skim milk media fed-batch bioprocess were utilized to test the immunomodulatory effect of *L. rhamnosus*. The fermentation harvest was centrifuged at 3360×*g* for 10 min, the pellet was collected and washed two times with distilled water before being resuspended to a final concentration of 25 mg/ml. These samples containing *L. rhamnosus* were added to a final concentration of 2.5 mg/ml to the 96-well plate containing 250 μL of J774 cells in a concentration of 10^6^ cells/ml and incubated at 37 °C in a humidified, 5% CO2 atmosphere for 1 h. Afterwards, lipopolysaccharide (LPS) was added to relevant wells as a positive control for enzyme-linked immunosorbent assay (ELISA) and dimethyl sulfoxide (DMSO) (Merck, New Jersey, USA) which was added as a negative control for 3-(4,5-dimethylthiazol-2-yl)-5-(3-carboxymethoxyphenyl)-2-(4-sulfophenyl)-2H-tetrazolium (MTS) assay. This plate was incubated at 37 °C in a humidified, 5% CO2 atmosphere for 24 h and the supernatant was later utilized for ELISA while the adherent cells on the 96-well plate were used for MTS assay.

### 2.13 Enzyme linked immunosorbent assay (ELISA)

The concentration of cytokines interleukin (IL)-1β, IL-6, IL-10, and tumour necrosis factor (TNF)-α was determined by sandwich ELISA (R&D Systems, Minneapolis, USA). Capture antibody was diluted in phosphate-buffered saline (PBS) (Thermo Fisher Scientific, Massachusetts, USA) and 50 μL was added to a 96-well microtiter plate to coat it (before leaving it to incubate overnight at room temperature. A washing step was performed where the 96-well plates were emptied for liquid, washed three times in wash buffer and lastly, the plates were blotted on paper towel to remove any trace wash buffer. Reagent diluent (RD) (Thermo Fisher Scientific, Massachusetts, USA) was added to block the wells and incubated overnight at 4 °C. Plates were washed as per the previous washing step and 50 μL of standards and samples appropriately diluted in RD were added to each well and left to incubate overnight at 4 °C. The plates were washed again and 50 μL of biotinylated detection antibody diluted in RD was added to each well and left to incubate in the dark at room temperature for 2 h. Plates were then washed again and a 50 μL of 1:40 streptavidin-horse radish peroxidase (HRP) solution (R&D Systems, Minneapolis, USA) was added to each well and left to incubate in the dark at room temperature for 20 min. Plates were then washed and 50 μL of 3,3′,5,5′-tetramethylbenzidine (TMB) substrate (BD Biosciences, California, USA) was added to each well and left to incubate in the dark at room temperature for 20 min. The reaction was stopped by adding 2 N H_2_SO_4_ (Thermo Fisher Scientific, Massachusetts, USA) and the optical density of the samples was determined using VersaMax TM microplate reader (Bristol Myers Squibb, New York, USA) at 450 nm.

The results of the assay are presented as mean ± standard error of the mean, and the groups were compared by one-way ANOVA with Tukey post hoc test using Graphpad Prism version 9.5.1 (Graphpad, California, USA).

### 2.14 MTS cell viability assay

To determine the cell viability an MTS assay was carried out using the CellTiter 96® AQueous One Solution Cell Proliferation Assay (Promega, Wisconsin, USA). 100 μL of fresh BMEM media was added to the previously seeded 96-well plates and 20 μL of CellTiter 96® Aqueous One Solution was added to each well before being incubated at 37 °C in a humidified 5% CO2 atmosphere for 3 h. The optical density of the samples was determined using VersaMax TM microplate reader at 490 nm.

## 3 Results

In this work, ANNs were used to accurately predict biomass and growth rate in bioprocesses. Biomass estimation is an important aspect of process control in bioprocesses, as it influences the productivity and quality of the final product (Ehgartner et al., 2017). As an example, the implementation of an ANN saw the enhancement of 46% of lipopeptide production from *Bacillus megaterium* in one study (Dhanarajan et al., 2014). This work demonstrates the use of artificial neural networks (ANNs) in accurately predicting biomass and growth rate in bioprocesses. Biomass estimation is a critical factor in process control for bioprocesses as it influences the productivity and quality of the final product.

The results of this study demonstrate that ANNs can serve as reliable estimators of biomass and growth rate in bioprocesses, achieving high levels of accuracy and precision. These findings suggest that ANNs have the potential to significantly improve process control in bioprocesses, enabling more efficient and effective production of functional food ingredients.

### 3.1 Biomass prediction model development

**Fig. 2:**
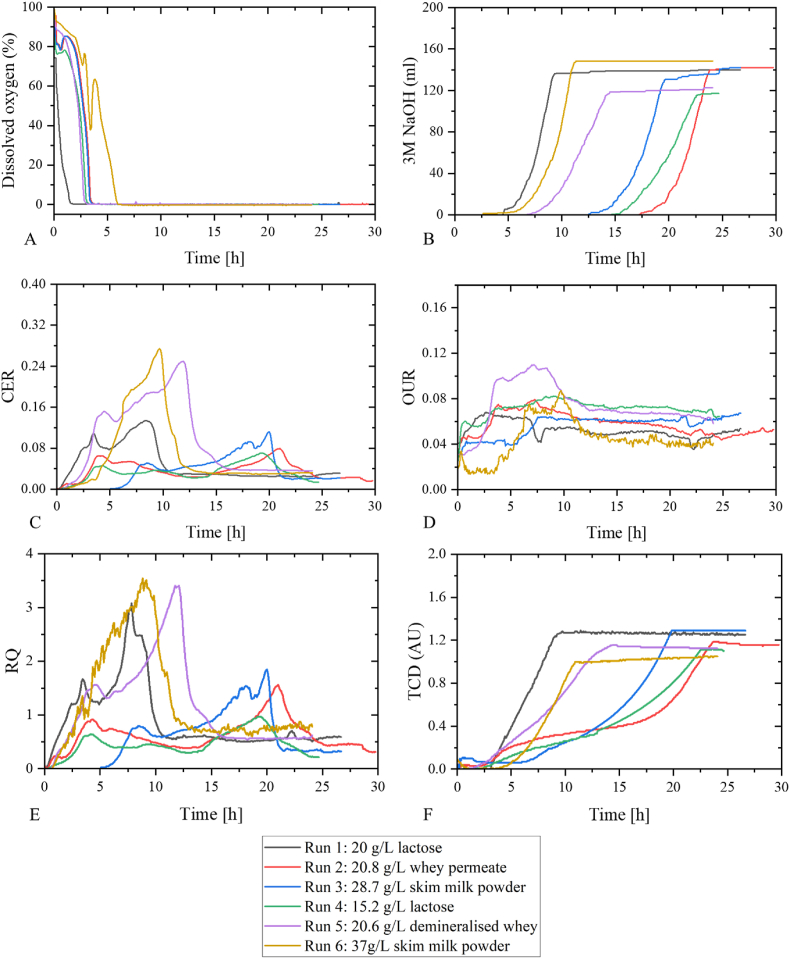
Profiles of bioprocess training data sets for ANN development (run 1–6.) (a) dissolved oxygen, (b) 3 M NaOH, c) CER, (d) OUR and (e) RQ, (f) TCD. In black: run 1 (20 g/L lactose), in red: run 2 (20.79 g/L demineralised whey), in blue: run 3 28.67 g/L skim milk powder, in green run 4: 15.23 g/L lactose, in purple: run 5 20.6 g/L whey permeate and in yellow 37 g/L skim milk powder. (For interpretation of the references to colour in this figure legend, the reader is referred to the Web version of this article.)

**Fig. 3:**
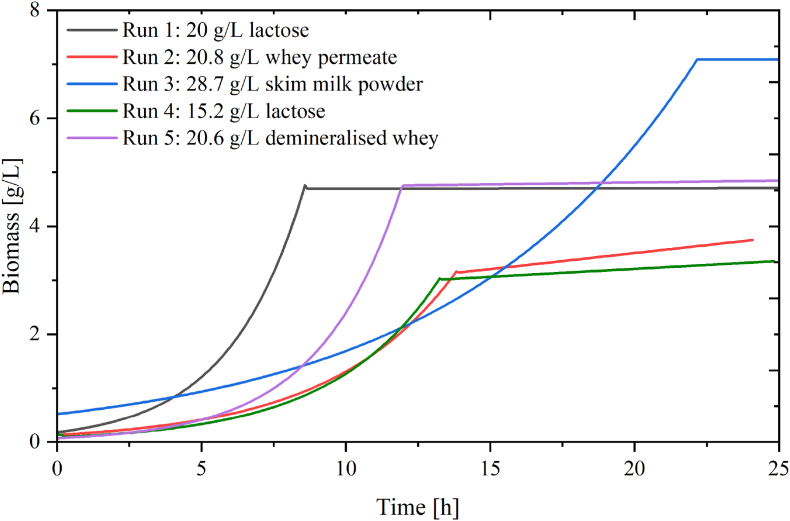
Growth profiles of datasets used for training the neural network. In black: run 1 (20 g/L lactose), in red: run 2 (20.79 g/L whey permeate), in blue: run 3 28.67 g/L skim milk powder, in green run 4: 15.23 g/L lactose and in purple: run 5 20.6 g/L demineralised whey. (For interpretation of the references to colour in this figure legend, the reader is referred to the Web version of this article.)

Six batch bioprocesses were cultivated with *L. rhamnosus* utilizing four different dairy-based substrates (lactose, whey permeate, demineralised whey and skim milk powder) of varying concentrations (Table 1). The data obtained from these bioprocesses were utilized to develop a three-layer feed-forward neural network for predicting biomass. The network was trained using the mean squared error as the optimization metric (Equation (12)), resulting in a predictive model with high accuracy as seen by the low RMSE (Table 2). Due to the variety of media components, several descriptive parameters on the composition of the media were included as input parameters to train the ANN together with parameters describing inoculum size, base addition, gas analyses and TCD (Fig. 2). The response variable, biomass, was modelled through the logistic equation (Equation (3)). The parameters X_m_, λ_x_ and V_x_. Were adjusted to minimize the error between the simulated and measured values. Fig. 3 illustrates the *L. rhamnosus* growth for the five bioprocesses used for training datasets as modelled through Equation (3) and highlights the variability in the media which is mainly caused by the different dairy substrate inputs.

**Table 2:** Model statistics of biomass prediction in batch bioprocess with values of hidden nodes between 1 and 15 and three different training engines Scaled conjugate gradient, Levenberg-Marquardt and Bayesian Regularization. Model performance was evaluated by RMSE and R^2^. The best performing model is highlighted in bold.

Statistics on model fit for three different training engines	Scaled Conjugate Gradient		Levenberg-Marquardt		Bayesian Regularization	
Hidden nodes	RMSE	R^2^	RMSE	R^2^	RMSE	R^2^
1	2.23	0.95	1.29	0.93	1.29	0.93
2	1.81	0.97	0.83	0.96	1.83	0.89
3	3.18	0.96	1.22	0.93	1.07	0.95
4	1.76	0.96	0.94	0.96	1.32	0.93
5	3.17	0.96	1.35	0.93	1.89	0.90
6	1.01	0.96	1.11	0.95	1.63	0.90
7	1.41	0.95	2.17	0.98	1.39	0.92
8	1.02	0.97	**0.74**	**0.98**	1.32	0.92
9	2.32	0.96	1.21	0.94	0.86	0.96
10	0.86	0.98	1.18	0.95	1.05	0.95
11	1.76	0.96	1.10	0.95	1.00	0.95
12	1.46	0.96	1.16	0.94	1.32	0.93
13	1.19	0.97	1.32	0.92	1.03	0.95
14	1.14	0.94	1.00	0.96	0.98	0.96
15	2.30	0.97	1.33	0.94	1.14	0.94

**Fig. 4:**
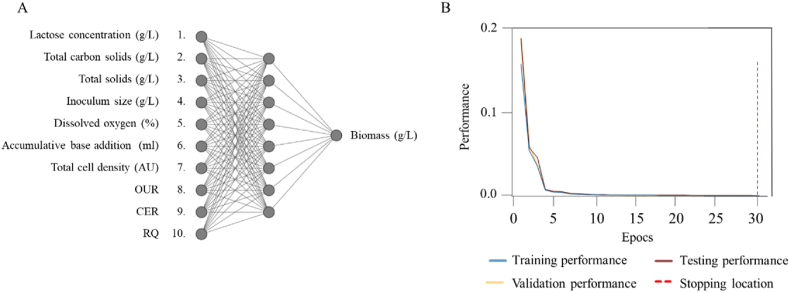
A) Topology of the neural network of biomass prediction in batch bioprocess, B) Training performance of the batch bioprocess data. In blue: training performance, in yellow: validation performance, in brown: testing performance, in red: stopping location. (For interpretation of the references to colour in this figure legend, the reader is referred to the Web version of this article.)

Three different training algorithms were employed and the number of hidden nodes varied between 1 and 15. The root mean squared error (RMSE) and the coefficient of determination (R^2^) were used as evaluation metrics to assess the performance of the predictive models. After conducting a comprehensive analysis of the different models, the best predictive model was found to have been obtained using the Levenberg-Marquardt algorithm with 8 nodes in the hidden layer. The neural network architecture with the ten inputs, 8 hidden nodes and a single biomass output is shown in Fig. 4 a.

This model achieved an RMSE value of 0.74 and an R2 value of 0.98 (Table 2). The model was trained using 5 iterations with a training goal of 0.001 at epoch 30 (Fig. 4b).

Upon selection of the best algorithm and ANN topology, the performance of the dataset was evaluated. The training samples had an RMSE of 0.016 and an R^2^ of 0.996, the validation samples had an RMSE of 0.018 and an R^2^ of 0.995, the testing samples had an RMSE of 0.022 and an R^2^ of 0.993 and finally, the total dataset had an RMSE value of 0.016 and an R^2^ of 0.995.

**Fig. 5:**
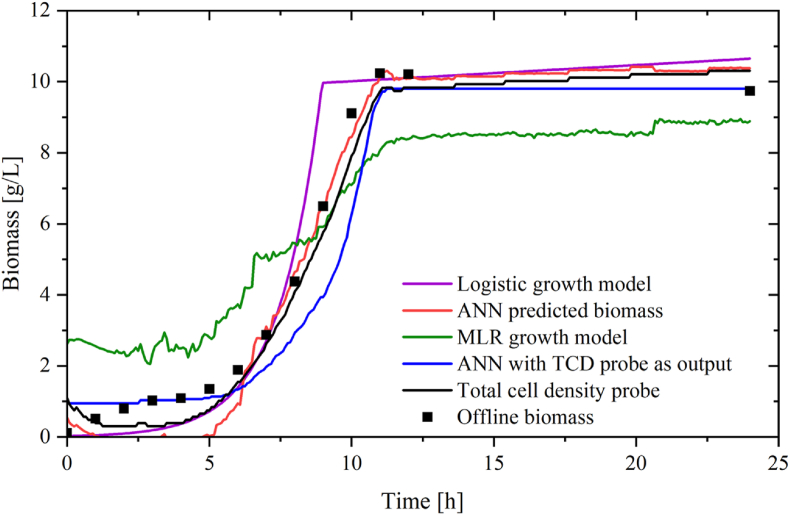
Biomass production in batch bioprocess. In purple: mechanistic biomass, in red: ANN predicted biomass, in green: biomass modelled through a multiple linear regression model, in blue: ANN with NIR as output factor, in black: reconciled total cell density probe, NIR signal, ■: off-line biomass. (For interpretation of the references to colour in this figure legend, the reader is referred to the Web version of this article.)

An unseen “testing dataset” (run 6, Table 1) was used to evaluate model performance. The ANN prediction was then compared with offline biomass measurement. Alternative estimations of biomass were developed as a comparison to the ANN model. Firstly, the data obtained from the TCD probe after calibration and data reconciliation were plotted. Secondly, the biomass was estimated using the logistic growth equation (Equation (3)) which was obtained by fitting the experimental offline samples to the logistic equation using the mean squared error as the optimization metric was included. A multiple linear regression (MLR) growth model based on the datasets from runs 1–7 was developed. These biomass estimations and models were evaluated by comparison to offline CDW samples taken hourly during the 12-h lag and exponential phase, as well as at the final time point of 24 h (Fig. 5Fig. 5). Furthermore, a neural network was created using data from the TCD probe as the output, while the input variables used in this neural network were otherwise similar to those presented in Figure. This second ANN had the output unit as AU and the output, therefore, needed calibration to biomass in g/L post-analysis.

Each of the five models was capable of predicting a cell biomass profile in line with offline samples (Fig. 5). The logistic model (3) underestimated biomass in the early lag phase while it overestimated the growth during the exponential phase and enters the log phase approximately 2 h before the experimental offline data. The TCD probe appears to represent the biomass values close to the off-line values, with only a slight underestimation. The major disadvantage of cell monitoring through this probe is however that the displayed signal is only obtained after substantial data reconciliation in the post-processing phase that includes media-dependent calibrations and filtering to obtain a representative curve. The ANN predicted biomass (in blue) was not quite capable of capturing the trend of growth during the early hours of fermentation in the log phase, but accurately estimated the growth when entering the exponential phase from 6 h until the end of fermentation when compared to the offline data. The datasets used to train the ANN were used to create an MLR model that was analysed using ANOVA. This model revealed that total carbon solids and OUR were not significant model terms and the following equation was produced:

Biomass=1.22−0.25*A+0.07*B+11.78*C−0.02*D+0.01*E+2.29*F+8.94*G−1.01*H

Where A is lactose concentration, B is total solids, C is inoculum size, D is dissolved oxygen, E is accumulative base addition, F is TCD probe, G is CER and H is RQ. As can be seen in Fig. 5 this model is capable of capturing the sigmoid profile of the growth but is otherwise poor at modelling the growth of *L. rhamnosus*. As both total carbon solids and OUR were revealed to be non-significant model terms, a third ANN was attempted without these inputs, but it was not possible to obtain a satisfactory prediction model without these factors (data not shown).

A secondary ANN for batch bioprocess was additionally developed, replacing biomass calculated by the logistic equation with the signal from the TCD probe. This model was developed with the scaled conjugate gradient algorithm with 9 hidden nodes (R^2^ = 0.95 and RMSE = 0.074). The limitations of this way of modelling are additionally the need for media-dependent calibration models making this model in the postprocessing phase, resulting in an ANN that is specific and not able to make general biomass predictions.

**Fig. 6:**
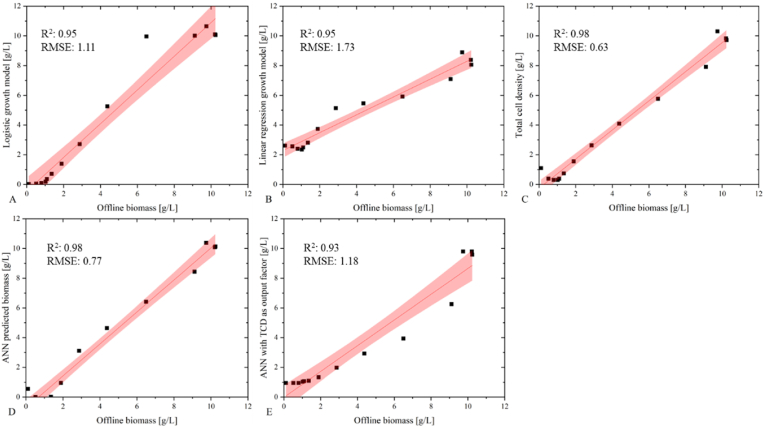
Actual vs. predicted plots of offline biomass and the five biomass models for batch bioprocess: A) logistic growth model, B) reconciled biomass measured by TCD probe, C) ANN predicted biomass and D) MLR growth, E) ANN predicted biomass with TCD as training response parameter model 95% confidence intervals are highlighted in red. (For interpretation of the references to colour in this figure legend, the reader is referred to the Web version of this article.)

Fig. 6 illustrates the performance of various prediction models and compares them to off-line cell dry weight measurements. Model validation plots of the logistic and multiple linear regression models can be seen in Fig. 6a and Fig. 6b. The logistic growth model shows a decent linear relationship with the offline biomass with an R^2^ value of 0.95 and an RMSE of 1.11, whereas the RMSE value of 1.73 supports the poor prediction ability of the multiple linear regression model in Fig. 6. Fig. 6c and d illustrate the relationship between offline biomass and the TCD probe and offline biomass and ANN predicted biomass, respectively. Both models display good correlation models with narrow confidence intervals and small RMSE values of 0.57 and 0.63 as well as high R^2^ values of 0.98. Finally, the ANN with TCD as its output showed a linear correlation with an R^2^ value of 0.93 and an RMSE of 1.18, Fig. 6 e.

**Fig. 7:**
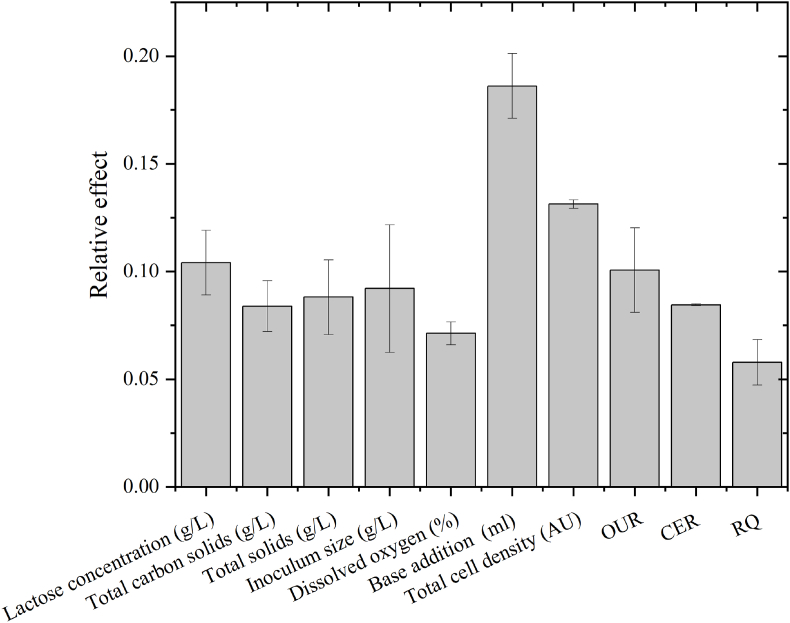
Relative effect of neural network inputs for biomass prediction in batch bioprocess. Error bars represent the average standard error for three runs.

The relative sensitivity of neural network inputs was calculated according to (Chellam, 2005). The effect of each input will vary with each neural network run, as the initial neural network weights are different for each training. Fig. 7 depicts the mean of three neural network training with the structure shown in Fig. 4A. Addition of NaOH is the input variable with the largest effect on the output (18.6%) followed by total cell density measurements (13.1%).

### 3.2 ANN prediction of fed-batch biomass

**Fig. 8:**
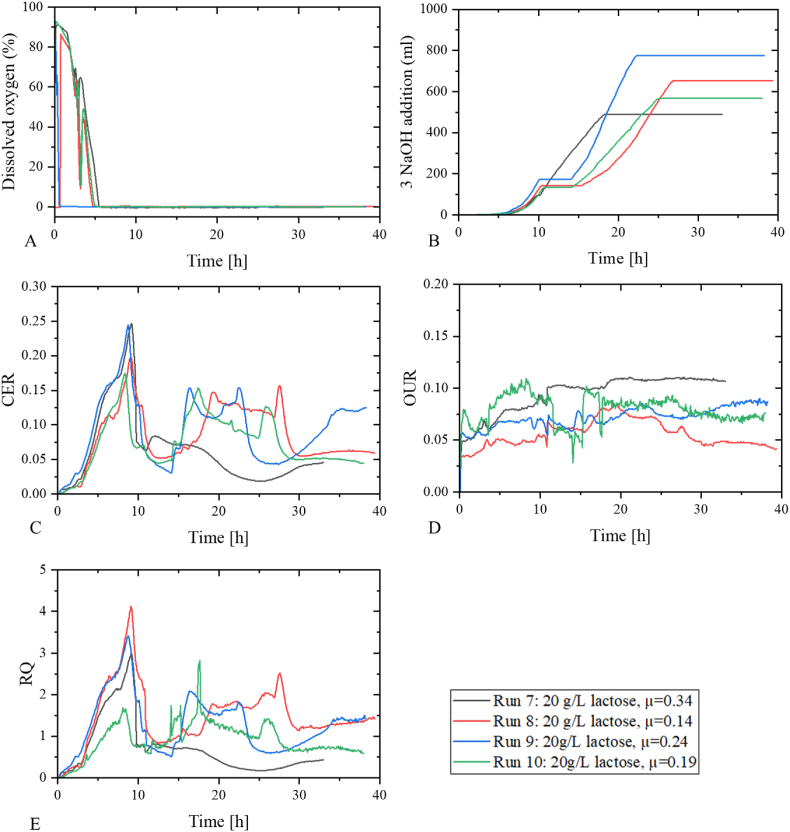
Profiles of input data of the ANN. a) dissolved oxygen, b) 3 M NaOH, c) CER, d) OUR and e) RQ. In black: run 7 (20 g/L lactose, μ-setpoint 0.34), in red: run 8 (20 g/L lactose, μ-setpoint 0.34), in blue: run 9 (20 g/L lactose, μ-setpoint 0.34). (For interpretation of the references to colour in this figure legend, the reader is referred to the Web version of this article.)

The datasets of four cultivations of *L. rhamnosus* in a skim milk powder-based media (Table 1, run 9–12) with an SMP feed with lactose concentrations of 100 g/L were applied in the development of an ANN for the prediction of biomass in fed-batch. The feed rates were determined using Equation (2) and with μ-setpoints of 0.14, 0.19, 0.24 and 0.34. The profiles of dissolved oxygen, the addition of 3 M NaOH, OUR, CER and RQ that were used and included in the ANN architecture can be seen in Fig. 8.

**Fig. 9:**
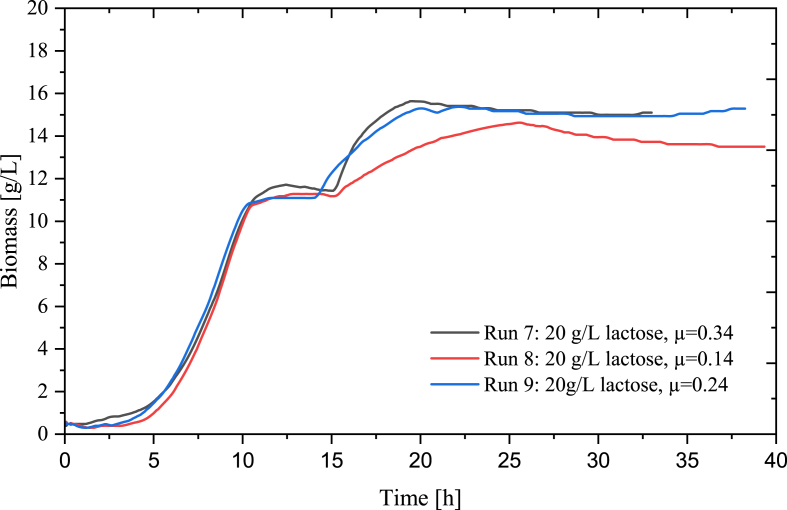
Growth profiles of datasets as measured by the TCD probe used for training the neural network. In black: run 7 (20 g/L lactose, μ-setpoint 0.34), in red: run 8 (20 g/L lactose, μ-setpoint 0.34), in blue: run 9 (20 g/L lactose, μ-setpoint 0.34). (For interpretation of the references to colour in this figure legend, the reader is referred to the Web version of this article.)

The growth profiles of the three fed-batch bioprocesses utilized in the training datasets can be seen in Fig. 9.

**Fig. 10:**
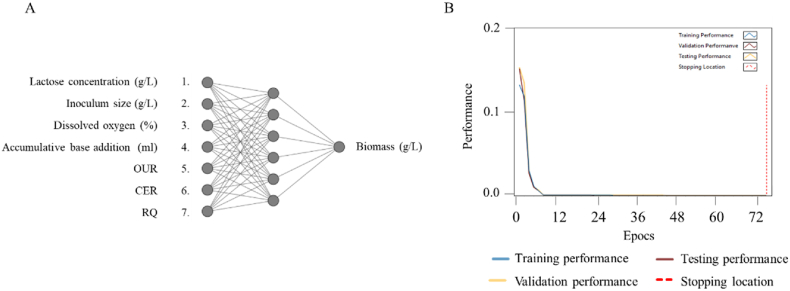
A) Topology of the neural network of biomass prediction in fed-batch bioprocess. B) Training performance of the fed-batch bioprocess data. In blue: training performance, in yellow: validation performance, in brown: testing performance, in red: stopping location. (For interpretation of the references to colour in this figure legend, the reader is referred to the Web version of this article.)

The network architecture and input parameters used for the development of the ANN for biomass prediction during fed-batch can be seen in Fig. 10. The best predictive model for biomass in the fed-batch phase was found using the Levenberg-Marquardt algorithm. Due to less complexity in the input layer, the best model was found with 6 hidden nodes and yielded an RMSE value of 0.40 and an R^2^ value of 0.99 (Table 3). These values were obtained using 5 training iterations with a training goal of 0.001 at epoch 67 (Fig. 10).

**Table 3:** Model statistics of biomass prediction in fed-batch bioprocess with values of hidden nodes between 1 and 15 and three different training engines: Scaled conjugate gradient, Levenberg-Marquardt and Bayesian regularization. Model performance was evaluated by RMSE and R^2^. The best performing model is highlighted in bold.

Statistics on model fit for three different training engines for fed-batch	Scaled Conjugate Gradient		Levenberg-Marquardt		Bayesian Regularization	
Hidden nodes	RMSE	R^2^	RMSE	R^2^	RMSE	R^2^
1	0.72	0.99	0.41	1.00	0.63	−0.15
2	1.05	0.96	0.84	0.99	0.49	0.82
3	0.95	1.19	0.73	0.99	0.48	−0.07
4	1.23	0.98	0.63	0.99	0.80	0.47
5	1.06	0.97	0.79	0.99	0.41	0.13
6	0.95	0.98	**0.40**	**0.99**	0.79	0.57
7	1.82	0.95	0.48	0.99	0.89	0.30
8	2.14	0.92	0.45	0.99	0.89	0.59
9	1.12	0.97	0.54	0.99	0.89	0.59
10	1.24	0.97	0.94	0.99	0.84	0.36
11	1.19	0.98	0.68	0.99	0.72	0.52
12	2.21	0.87	0.76	0.98	0.66	0.56
13	1.77	0.96	0.65	1.00	0.61	0.47
14	2.14	0.96	0.60	1.00	0.65	0.39
15	1.81	0.95	0.84	0.99	0.67	0.66

The training data set had an RMSE of 0.001 and an R^2^ of 0.99, the validation sub-set had an RMSE of 0.011 and an R^2^ of 0.999, the testing sub-set had an RMSE of 0.012 and an R^2^ of 0.99 and the total dataset had an RMSE value of 0.011 and an R^2^ of 0.99.

After the selection of the appropriate model, a test dataset unknown to the model of a fed-batch run utilizing skim milk powder as its dairy-based substrate was tested.

**Fig. 11:**
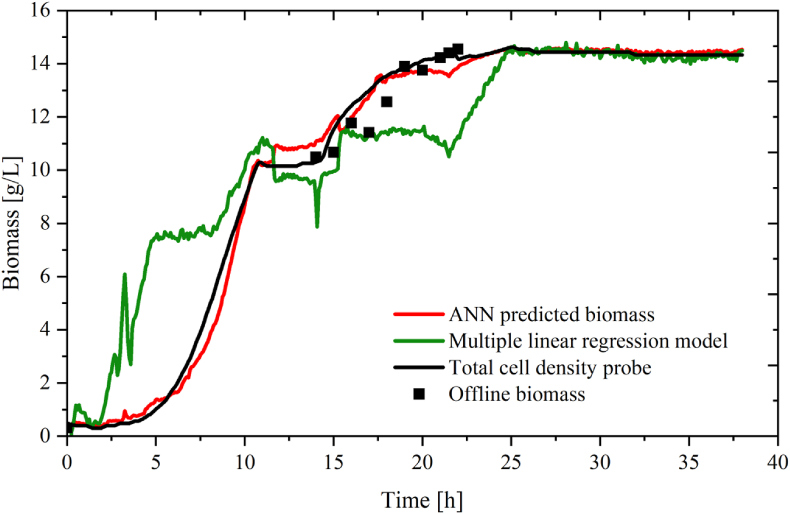
Biomass production during fed-batch. In red: ANN predicted biomass, in green: multiple linear regression model, in black: TCD probe, ■: off-line biomass. (For interpretation of the references to colour in this figure legend, the reader is referred to the Web version of this article.)

To assess the accuracy of the prediction, offline samples for CDW determination were collected at hourly intervals throughout the fed-batch process and were compared to the data obtained from the TCD, as illustrated in Fig. 11. The ANN biomass model seems to be very similar to the reconciled biomass profile obtained from the TCD probe. The ANN model also compared well to the offline samples. The largest variation between the TCD and the ANN-predicted biomass can be observed during the feed phase of the process which is from 15 to 22 h. With the addition of the feed, this phase is characterised by being more dynamic, which can be seen by the higher level of deviation between measured and predicted values. The fed-batch fermentation process allows for the controlled introduction of nutrients, which promotes the growth and metabolism of *L*. *rhamnosus*. This dynamic environment leads to variations in the data compared to batch fermentation, where the nutrients are added all at once at the beginning of the process. Similar to what was observed in batch bioprocess in Fig. 5, the multiple linear regression model was not capable of accurately capturing the biomass production in fed-batch (Fig. 11).

The performance of the biomass estimation by the TCD probe, the ANN and the multiple linear regression model was evaluated against the offline biomass (Fig. S2). Both Fig. S2 a and Fig. S2 b illustrate the capability of the reconciled TCD probe data and the ANN to accurately predict biomass as seen by a good linear fit, a low RMSE values of 0.62 and 0.76 and high R^2^ values of 0.98 and 0.97 obtained for the TCD probe and ANN respectively. Moreover, it is noteworthy that the ANN model was able to accurately predict biomass during fed-batch, despite not containing any direct measurements of biomass as input parameters. The poor predictive power of the multiple linear regression model can be seen by the lower R^2^ and high RMSE of 2.39 (Figure S2c).

The MLR model that consisted of data from all four fed-batch bioprocesses was furthermore used to assess the effectiveness of the input variables to predict the response variable biomass. The model was evaluated with ANOVA and showed that the model was statistically significant (p < 0.0001). The individual impact of each input variable was evaluated by examining their p-values, and all six variables had a significant impact on the response variable (p < 0.05).

**Fig. 12:**
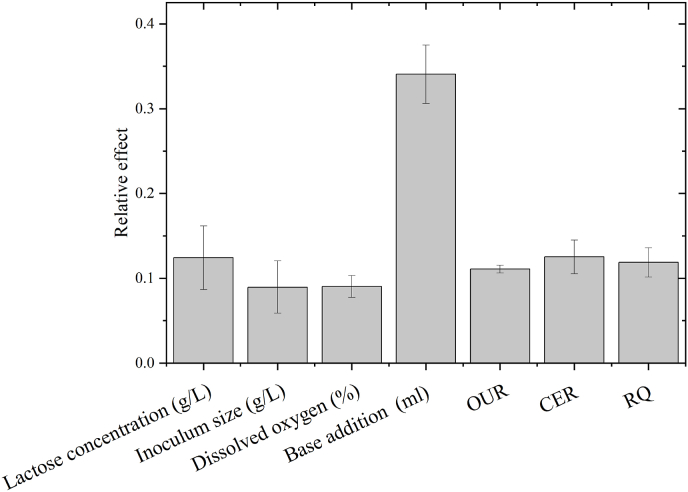
Relative effect of neural network inputs for biomass prediction in fed-batch bioprocess. Error bars represent the average standard error for three runs.

The relative effect of the input parameters is depicted in Fig. 10A for the neural network predicting biomass in fed-batch was also assessed (Equation (15)). Similar to the neural network developed for prediction of biomass in batch bioprocess, the addition of base had the highest relative effect of 34% when predicting biomass in fed-batch with all other inputs having a far lower effect between 9% and 12% (Fig. 12). This high effect of base addition is most likely due to the fermentative link between bacterial growth and production of lactic acid which is furthermore supported by the data presented in Fig. 8B, where the addition of base follows a very similar trend to that of bacterial growth.

### 3.3 ANN prediction of growth rate

**Fig. 13:**
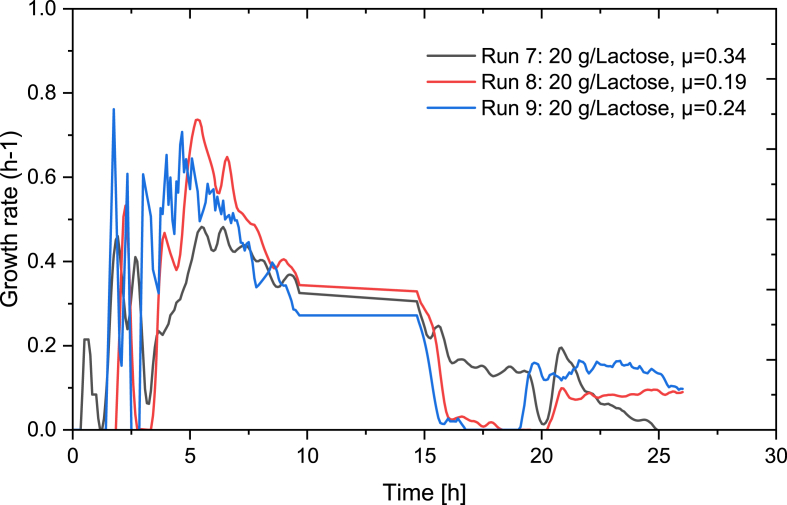
Growth rate profiles of datasets used for training the neural network. In black: run 7 (20 g/L lactose, μ-setpoint 0.34), in red: run 8 (20 g/L lactose, μ-setpoint 0.34), in blue: run 9 (20 g/L lactose, μ-setpoint 0.34). (For interpretation of the references to colour in this figure legend, the reader is referred to the Web version of this article.)

The development of an ANN for predicting the growth rate in *L. rhamnosus* was based on the same four datasets utilized for the prediction of biomass in fed-batch bioprocess but had included the additional input of ANN-predicted biomass. The response (growth rate) was calculated based on the biomass data from the TCD probe, with the profiles of the three datasets seen in Fig. 13.

**Fig. 14:**
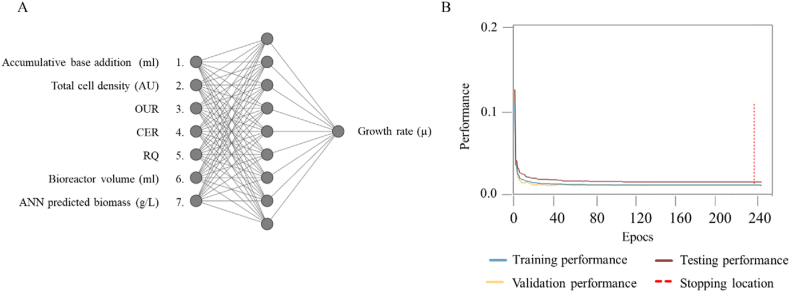
A) Topology of the neural network of growth rate prediction in fed-batch. B) Training performance of the growth rate for the fed-batch bioprocess data. In blue: training performance, in yellow: validation performance, in brown: testing performance, in red: stopping location. (For interpretation of the references to colour in this figure legend, the reader is referred to the Web version of this article.)

Three machine learning algorithms were employed, with the number of hidden nodes varying from 1 to 15. These were evaluated by the same metrics previously described. Analysis of the models developed was performed, and the most effective predictive model utilized the scaled conjugate gradient algorithm with 10 hidden nodes (Fig. 14).

This model achieved an RMSE value of 0.082 and an R^2^ value of 0.89 (Table 4). The model was trained using 5 iterations with a target training error of 0.001 at epoch 250 (Fig. 14b).

**Table 4:** Model statistics of growth rate in fed-batch bioprocess with values of hidden nodes between 1 and 15 and three different training engines: Scaled conjugate gradient, Levenberg-Marquardt and Bayesian regularization. Model performance was evaluated by RMSE and R^2^. The best performing model is highlighted in bold.

Statistics on model fit for three different training engines for fed-batch	Scaled Conjugate Gradient		Levenberg-Marquardt		Bayesian Regularization	
Hidden nodes	RMSE	R^2^	RMSE	R^2^	RMSE	R^2^
1	0.109	0.77	0.09	0.75	0.11	0.75
2	0.086	0.90	0.13	0.50	0.29	0.38
3	0.116	0.76	0.25	0.24	0.23	0.28
4	0.083	0.89	0.14	0.46	0.26	0.41
5	0.098	0.83	1.50	0.39	0.17	0.45
6	0.103	0.83	0.84	0.38	0.34	0.08
7	0.086	0.86	0.33	0.04	0.39	0.16
8	0.091	0.90	0.44	0.25	0.55	0.28
9	0.094	0.89	1.67	0.12	0.60	0.06
10	**0.081**	**0.90**	0.61	0.21	0.83	0.21
11	0.083	0.87	0.88	0.21	0.58	0.06
12	0.085	0.90	0.44	0.01	0.46	0.05
13	0.091	0.86	0.66	0.34	0.52	0.08
14	0.103	0.847	0.66	0.34	0.88	0.23
15	0.122	0.729	0.34	0.06	0.96	0.12

The training samples had an RMSE of 0.071 and an R^2^ of 0.69, the validation samples had an RMSE of 0.082 and an R^2^ of 065, the testing samples had an RMSE of 0.081 and an R^2^ of 0.63 and the total dataset had an RMSE value of 0.074 and an R^2^ of 0.68.

**Fig. 15:**
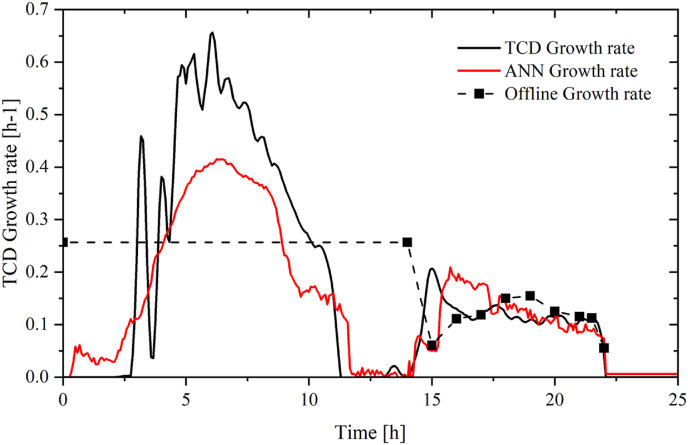
Growth rate during fed-batch. In grey: TCD growth rate, in red: ANN predicted growth rate ■: off-line growth rate. (For interpretation of the references to colour in this figure legend, the reader is referred to the Web version of this article.)

The growth rate graph in Fig. 15 illustrates the cell growth during a bioprocess with feed from hours 14–22. In the batch bioprocess, the initial growth progressively increases until it reaches a maximum value at approximately 7 h into the process. Subsequently, the growth rate declines and eventually reaches a plateau as the cells enter the stationary phase. In the fed-batch bioprocess, the growth rate increases as soon as feeding commences. However, the peak value is significantly smaller compared to the peak observed during the batch bioprocess and remains relatively constant throughout the feed phase.

During the fed-batch fermentation process presented in Fig. 15, the feed rate was introduced at an exponential rate with a μ setpoint of 0.19 in order to support the growth of the cells. However, this increase in the feed rate was not reflected in the growth rate, which appears to be decreasing over time and had an average experimental value of 0.13. This signifies the importance of implementing model predictive control to be able to enact change on the system.

This discrepancy may be due to a variety of factors, including the depletion of certain essential nutrients and salts during the initial growth phase in the batch process. This lack of certain salts such as magnesium, manganese, potassium, iron or other essential nutrients during the fed-batch phase may cause the growth rate to decline, even though the feed rate is being increased exponentially (Macedo et al., 2002; Gamar et al., 1997; Macleod and Snell, 1947).

**Fig. 16:**
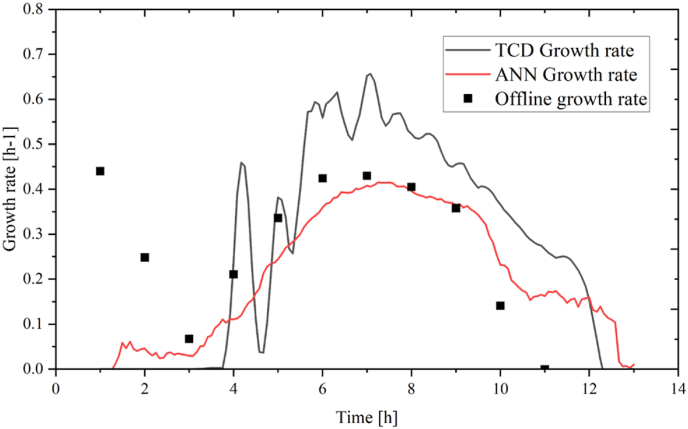
Growth rate during batch. In grey: TCD growth rate, in red: ANN predicted growth rate ■: off-line growth rate. (For interpretation of the references to colour in this figure legend, the reader is referred to the Web version of this article.)

The ANN growth rate model was applied to the batch data from run 6. It was seen to have a slightly higher accuracy in prediction than compared with TCD monitoring. Notably, it seemed to have a lower sensitivity to noise and did not have the same issues of overestimation of higher growth rate conditions (>0.4 h^−1^) as seen with the monitoring of the TCD probe (Fig. 16).

**Fig. 17:**
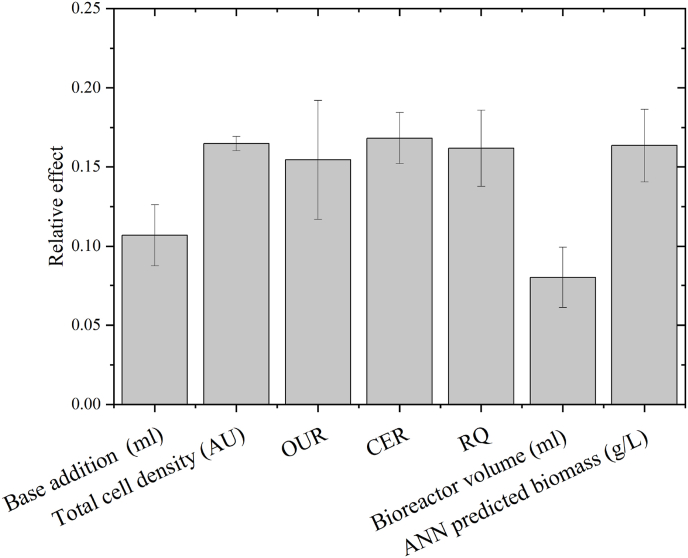
Relative effect of neural network inputs for growth rate prediction in fed-batch bioprocess. Error bars represent the average standard error for three runs.

Model performance was again evaluated by examining the actual vs. predicted plots of the data (S3). However, these plots only show the actual vs. predicted of growth rate during the batch phase as the data from the fed-batch had very small magnitudes of variation that resulted in poor linearity and inaccurate representation of data in actual vs. predicted plots.

Both of the actual vs. predicted plots presented in Fig. S3 Show strong linear relationships between offline growth rate and ANN growth rate and TCD growth rate, respectively. The lower RSME value of Fig. S3 a compared to Fig. S3 b does however indicate that the ANN predicted growth rate is better at capturing the trends observed in the offline data. To evaluate the significance of each of the inputs a nonlinear regression model was attempted, but with the complexity of the data, it was not possible to adequately generate a model.

The relative effect of each input parameter could however be assessed through Equation (15).

As opposed to the neural networks predicting biomass, no inputs had a noticeably higher effect on the output variable growth rate. Both total cell density (16.5%), OUR (15.5%), CER (16.8), RQ (16.2) and ANN predicted biomass (16.4%) have a similar relative effect on the output variable. The input with the lowest relative effect is bioreactor volume (8%) which is also the only input parameter that does not describe either bacterial growth or the current state of the cells.

### 3.4 The immune response of L. rhamnosus cell samples

The immune response of the *L. rhamnosus* biomass produced during the bioprocess of furthermore investigated for its ability to elicit an immune response to validate its functionality as a health-promoting food ingredient (see Fig. 17).

**Fig. 18:**
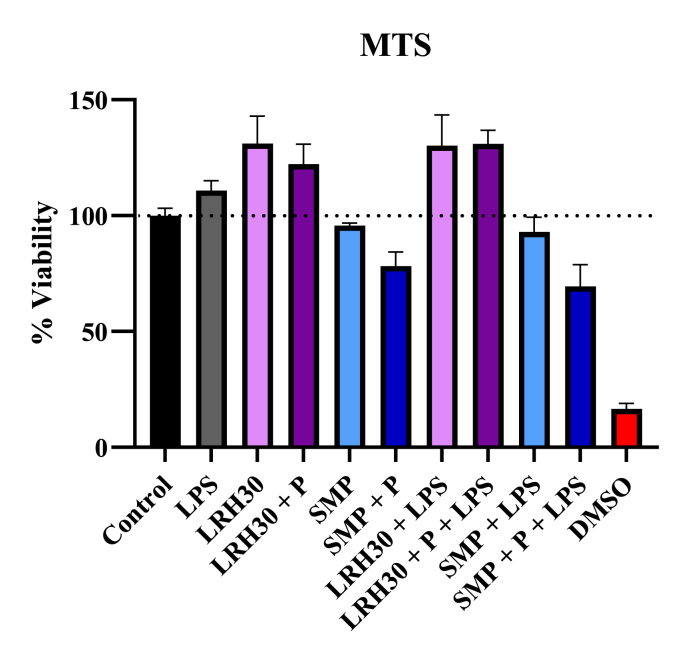
J774 viability after treatment with *L. rhamnosus* samples and SMP with and without protease and LPS stimulation. DMSO was included as a positive control.

The macrophage viability was assessed through an MTS assay when treated with *L. rhamnosus* (LRH30) and reconstituted skim milk (SMP) samples. The cell viability of the J774 cells appears to increase, exceeding the values of control, when treated with LRH30 both with and without protease and LPS stimulation. This positive effect on cell proliferation is evidenced by the viability which varied between 122 and 130% for all LRH30 samples. SMP samples did however impact the macrophage viability, as it slightly decreased for SMP (95.6%) and SMP with LPS (92.9%). This viability was further decreased when the SMP was treated with protease (P) as the measured viability reduced to 78.2% for SMP with protease and to 69.4% for SMP with protease and LPS stimulation (Fig. 18).

**Fig. 19:**
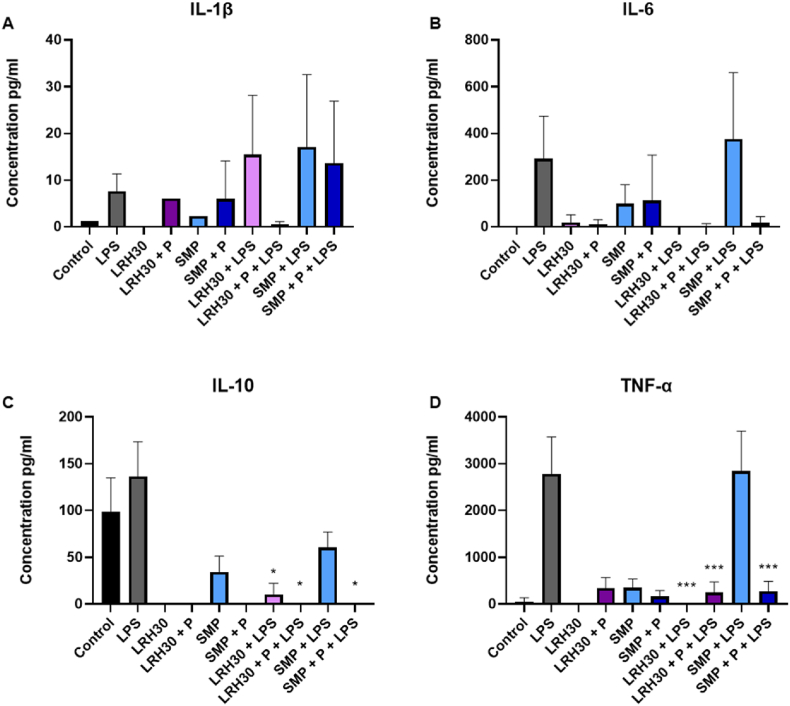
Secretion of IL-1β, IL-6, Il-10 and TNF-α by cells treated with *L. rhamnosus* samples and SMP with and without protease and LPS stimulation. Significance p < 0.05 *, p < 0.01**, p < 0.001 ***.

The ability of *L. rhamnosus* to elicit an immune response was then investigated by evaluating their effect on the excretion of cytokines by J774s. In this study, the inflammatory cytokines IL-1β, IL-6 and TNF-α were measured as well as the anti-inflammatory cytokine IL-10 using ELISA (Fig. 19). J774 cells were treated with samples of LRH30 and SMP with and without protease and with and without LPS stimulation (100 ng/ml). Since the cells were grown in SMP-based media, SMP was included in the assay together with the addition of protease to evaluate whether a possible effect was induced by cells or the media itself. The LRH30 sample showed a strong ability to decrease an anti-inflammatory response as a trend of reduction of levels of IL-6 was observed (Fig. 19b). Additionally, TNF-α was significantly reduced (p < 0.0001) by the addition of LRH30 (Fig. 19d), while the levels obtained for IL-1β were too low to be considered significant (Fig. 19a). The LRH30 sample did however also decrease the levels of IL-10 in a significant manner (p < 0.05), (Fig. 19c).

## 4 Discussion

The results of this study indicate a strong ANN predictive capacity for the biomass profiles of *L. rhamnosus* bioprocesses in both batch and fed-batch bioprocesses. Additionally, a satisfactory model for predicting the growth rate of *L. rhamnosus* **i**n fed-batch conditions was developed. These findings demonstrate the potential applications of predictive modelling in optimizing the cultivation of *L. rhamnosus*.

The ANN developed for the batch phase proved to have a better capability to accurately predict biomass than the raw data generated by the TCD probe. Several factors make the use of the TCD probe challenging for these bioprocesses including the media complexity, lack of homogeneity throughout the process, need for calibration and reconciliation and the lack of sensitivity (Cervera et al., 2009; Rinnan et al., 2009). The media in this study experiences an increased viscosity and generation of milk gels that interfere with the homogeneity of the fermentation broth and thereby disrupt the signal (Arnold et al., 2002).

The lack of sensitivity of the TCD probe is an additional problem that becomes highly evident in the growth rate model, where small changes to biomass concentration translate to high oscillations in the hereof-derived growth rate (Soons et al., 2008).

Lastly, the TCD probe requires calibration to relate the signal to actual biomass, and we have found that this calibration is highly dependent on the composition of the media and is therefore time-consuming.

TCD probes have however found application for the measurement of biomass and growth rate in other bacterial cultivations but here a defined media was applied and additional calibration and filtering were still needed to interpret the signal (Soons et al., 2008; Warth et al., 2010).

The mechanistic model used in this study was based on a logistic equation proposed by Balakrishnan et al. (2020). This model simulates the biomass of *Lactobacillus* independently of substrate consumption (Balakrishnan et al., 2020). While this model was able to provide good simulations of biomass in batch bioprocesses, its simplicity makes it inadequate for simulations of biomass in fed-batch processes. This is due to the inherent complexity of fed-batch processes, which involve the continuous addition of nutrients over time and the dynamic interactions between multiple variables. As such, more comprehensive models may be required to accurately simulate biomass in fed-batch processes. A far better understanding of the process kinetics and underlying biochemistry is therefore crucial in order to incorporate and formulate the complex interactions between the multiple variables for a mechanistic model (Del Rio-Chanona et al., 2019; Goudar, 2012). This inability to properly capture the trends in growth was also observed in the attempt to predict the biomass in batch bioprocess through MLR modelling. This is most likely due to the high correlation between certain variables that will not be captured by MLR as well as the lower capability of MLR to manage outliers when compared to ANN models.

One of the current limitations of the ANNs developed in this study exists in their simple architecture of the 3-layered models, which only includes one hidden layer. This may not provide sufficient capacity for the network to learn and properly represent the complexity of the data (Uzair and Jamil, 2020). Increasing the complexity of the ANN topology, for example, by adding additional hidden layers, may improve the performance of the network and enable it to more accurately represent the complexity of the data. However, it is important to carefully balance the complexity of the topology against the risk of overfitting, as increasing the complexity of the ANN can also increase the risk of poor generalization to new data (Caruana et al., 2000; Gavrilov et al., 2019). Another weakness is the limited number of datasets applied to train the model. This combined with the simple topology might cause challenges in properly recognizing the patterns of the growth rate model (Greener et al., 2022).

This work aimed to develop an ANN capable of predicting biomass and growth rate for implementation in fed-batch bioprocess optimization and control. The application of optimized and robust control systems through ANN modelling can lead to more efficient bioprocesses and ultimately higher volumetric productivity of *L. rhamnosus*. Previous studies have successfully implemented ANNs for online control of bioprocesses and hereby improved the yield and repeatability of processes (Kovárová-Kovar et al., 2000; Geethalakshmi et al., 2012; Lopez et al., 2020). This ANN development constitutes the basis of the implementation of a digital twin. Real-time data from the cultivations of *L. rhamnosus* can feed into the digital model in real-time, while the output from the digital model can be fed back into the physical process, thereby acting as a closed-loop model-based controller and creating a digital twin.

The ANNs predicting biomass and growth rate will be implemented as estimators for regulating the addition of feed to the bioprocess. This enables more precise control of bioprocess conditions and cell kinetics, reducing the risk of nutrient excess or depletion. The neural network models developed in this work are the start of a digital twin development for a *Lactobacillus* bioprocess. The models implemented online will enact change in real-time, utilizing feed-forward closed-loop control of the substrate of the bioreactor. This will be part of an overall system to monitor, fault find and optimize the process in real-time and offline.

The immunomodulatory effect observed in this work aligns with previous studies on the inhibition of the pro-inflammatory cytokines IL-6 and TNF-α by *L. rhamnosus* (Peña and Versalovic, 2003; Vargas García et al., 2015; Qi et al., 2020; Kim et al., 2006). Contrarily to the suppressive effect on anti-inflammatory cytokine IL-10 secretion found in this work, some studies have reported the stimulatory effect on the IL-10 secretion by *L. rhamnosus* (Vargas García et al., 2015; Jeffrey et al., 2020)*,* while others observed no effect on IL-10 secretion (Qi et al., 2020; Bleau et al., 2010).

One study compared the immunostimulatory effects of IL-6, IL-10 and TNF-α from two strains of *L. rhamnosus*, a low and a high exopolysaccharide (EPS)-producing strain. The effect here on cytokine secretion on macrophages by the EPS-producing strain of *L. rhamnosus* strains was similar to those observed in this study with low amounts of IL-6 and TNF-α secreted in the presence of *L. rhamnosus*, while also not seeing any stimulatory effect on IL-10 (Bleau et al., 2010). It has been suggested that *L. rhamnosus* has a production preference for immunosuppressive molecules (Peña and Versalovic, 2003) and that a low secretion of IL-10 is a general phenomenon for monocytes when exposed to gram-positive bacteria (Hessle et al., 2000).

## 5 Conclusion

The data from six batch bioprocesses and four fed-batch bioprocesses was successfully applied in the development and evaluation of two ANNs with the capability of predicting biomass in batch bioprocesses with varying media compositions and in predicting biomass in SMP fed-batch bioprocesses. The accuracy in predicting biomass exhibited by the ANNs predicting biomass indicated good potential for applications in bioprocess control.

An ANN with a satisfactory ability to predict the growth rate in fed-batch bioprocess was furthermore modelled, but additional research data and refinement of the ANN architecture might be needed to obtain a better accuracy of the predictions. The μ-setpoint of the fed-batch phase was 0.19, but the average value of μ during this window was only 0.13. This highlights the importance of applying the developed digital twin of the process in model predictive control, as better predictions and therefore better control of the actual process could be obtained.

This study highlights the advantages of using ANN modelling as a tool in bioprocess modelling, estimation and control by comparing it with conventional modelling approaches such as mechanistically modelled biomass and sensing technologies for biomass estimation. The health-promoting effects of *L. rhamnosus* were furthermore confirmed by its ability to lower the expression of the anti-inflammatory cytokines IL-6 and TNF-α.

As such, this work demonstrates the potential of bioprocess optimization through ANN modelling to obtain a higher production yield of valuable microbial products with important applications within the functional food industry.

## CRediT authorship contribution statement

**Helena Mylise Sørensen:** Conceptualization, Experimental work, Writing, Writing – review & editing. **David Cunningham:** Experimental work, Writing – review & editing. **Rengesh Balakrishnan:** Experimental work. **Susan Maye:** Writing – review & editing. **George MacLeod:** Writing – review & editing. **Dermot Brabazon:** Project administration, and, Funding acquisition, All authors have read and agreed to the published version of the manuscript. **Christine Loscher:** Writing – review & editing, Project administration, and, Funding acquisition. **Brian Freeland:** Conceptualization, Writing, Writing – review & editing, Project administration, and, Funding acquisition.

## Declaration of competing interest

The authors declare the following financial interests/personal relationships which may be considered as potential competing interests:Helena Mylise Sorensen reports financial support was provided by 10.13039/501100001602Science Foundation Ireland.

## Data Availability

The data that has been used is confidential.

## Footnotes

Appendix A Supplementary data to this article can be found online at https://doi.org/10.1016/j.crfs.2023.100593.
