# Supplementary material for: Steps toward a digital twin for functional food production with increased health benefits
Source: Curr Res Food Sci. 2023 Sep 26;7:100593. doi: 10.1016/j.crfs.2023.100593 (PMC10543970; doi:10.1016/j.crfs.2023.100593)
Supplement: Multimedia component 1 [file mmc1.docx]

Supplementary material: Steps Toward a Digital Twin for Functional Food Production with Increased Health Benefits


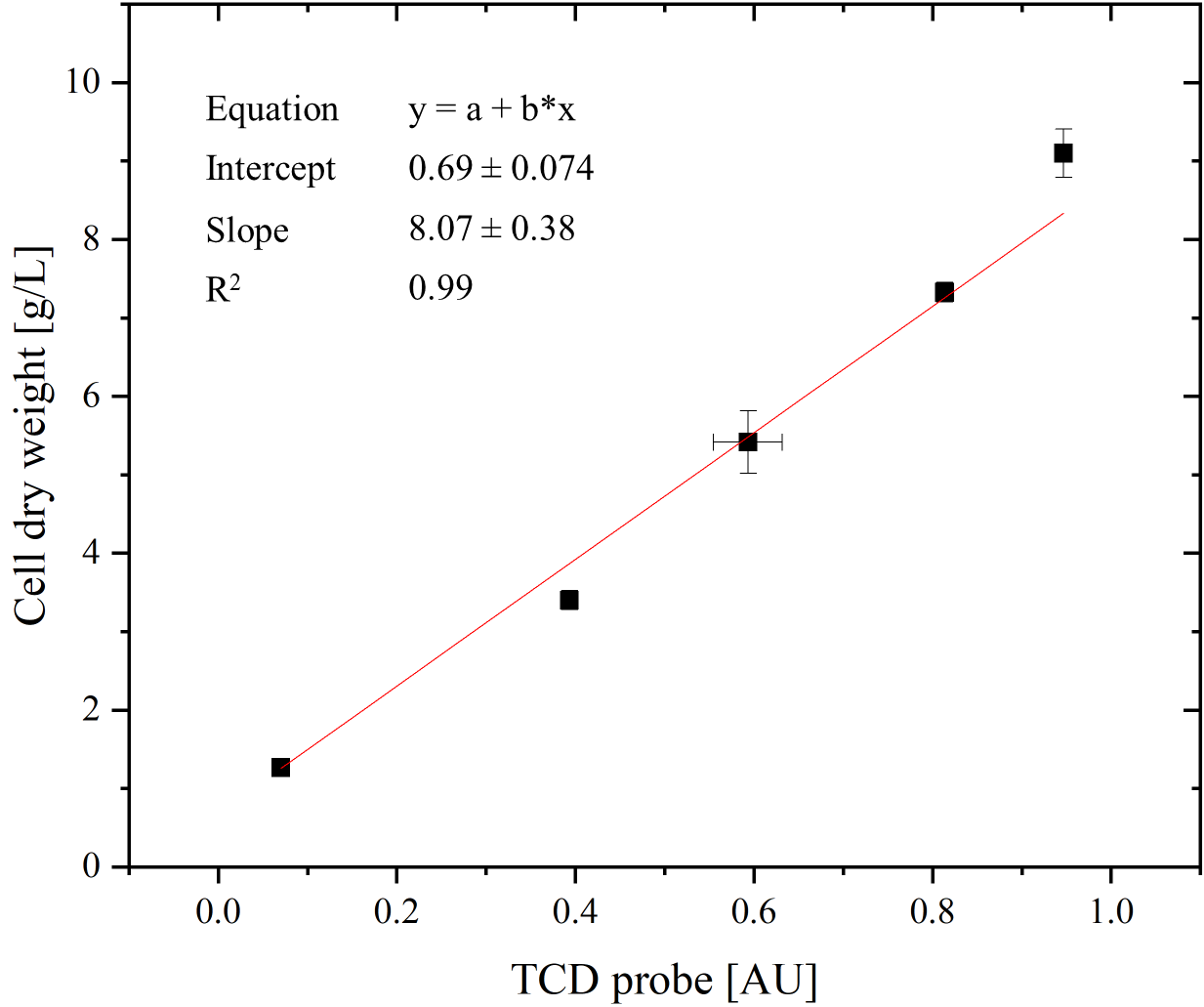


S1: Calibration curve for L. rhamnosus growtn in skim milk powder-based media .


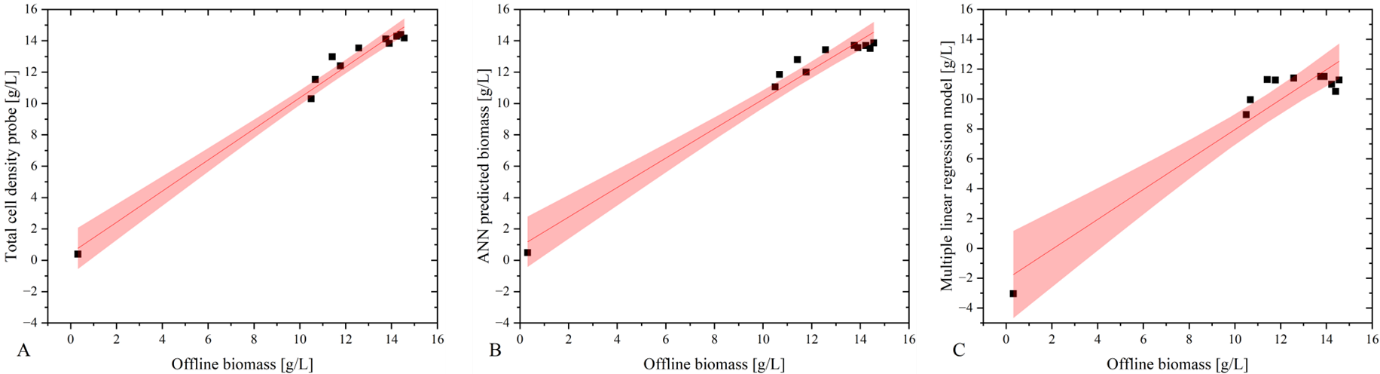


S2: Actual vs predicted plots of offline biomass and the two biomass models for fed-batch bioprocess: A) reconciled biomass measured by TCD probe and B) ANN predicted biomass and C) Multiple linear regression model. 95% confidence intervals are highlighted in red.


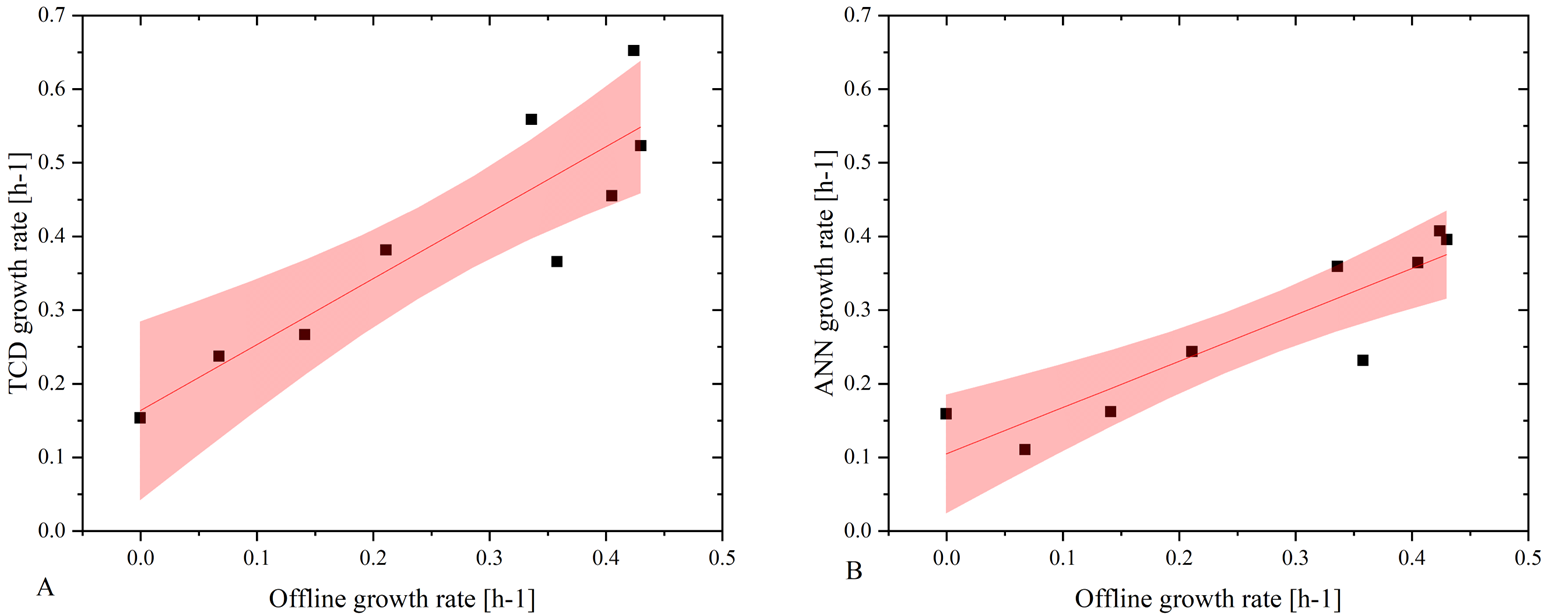


Figure S3: Actual vs. predicted plots of offline growth rate and the two growth rate models for the batch phase model: A) reconciled growth rate measured by TCD probe growth rate B) ANN predicted growth rate. 95% confidence intervals are highlighted in red.
